# Multi-Sensor Fusion: A Simulation Approach to Pansharpening Aerial and Satellite Images

**DOI:** 10.3390/s20247100

**Published:** 2020-12-11

**Authors:** Katarzyna Siok, Ireneusz Ewiak, Agnieszka Jenerowicz

**Affiliations:** Faculty of Civil Engineering and Geodesy, Military University of Technology, 00-908 Warsaw, Poland; ireneusz.ewiak@wat.edu.pl (I.E.); agnieszka.jenerowicz@wat.edu.pl (A.J.)

**Keywords:** spectral quality, simulation, pansharpening, multi-sensor fusion, aerial images, satellite imagery

## Abstract

The growing demand for high-quality imaging data and the current technological limitations of imaging sensors require the development of techniques that combine data from different platforms in order to obtain comprehensive products for detailed studies of the environment. To meet the needs of modern remote sensing, the authors present an innovative methodology of combining multispectral aerial and satellite imagery. The methodology is based on the simulation of a new spectral band with a high spatial resolution which, when used in the pansharpening process, yields an enhanced image with a higher spectral quality compared to the original panchromatic band. This is important because spectral quality determines the further processing of the image, including segmentation and classification. The article presents a methodology of simulating new high-spatial-resolution images taking into account the spectral characteristics of the photographed types of land cover. The article focuses on natural objects such as forests, meadows, or bare soils. Aerial panchromatic and multispectral images acquired with a digital mapping camera (DMC) II 230 and satellite multispectral images acquired with the S2A sensor of the Sentinel-2 satellite were used in the study. Cloudless data with a minimal time shift were obtained. Spectral quality analysis of the generated enhanced images was performed using a method known as “consistency” or “Wald’s protocol first property”. The resulting spectral quality values clearly indicate less spectral distortion of the images enhanced by the new methodology compared to using a traditional approach to the pansharpening process.

## 1. Introduction

Image data are a rich source of information about the surface of the Earth. They are widely used in many disciplines, from agriculture to state defense policy [1]. However, a single image is usually not enough to make a comprehensive analysis of the land cover. Due to design limitations, sensors operating at both satellite and aerial photography altitudes do not yield data with a high spatial and spectral resolution at the same time, or the cost of obtaining such data (particularly aerial) is too high. In order to carry out detailed studies of the Earth’s surface, it is necessary to have images of both high spectral and spatial quality [2]. Higher-quality data enable more accurate spatial analysis, thus improving the decision-making process. For long-term analyses, archival aerial photos play a crucial role. These are usually single-band or RGB images (red, green, and blue bands) that often are the only source of information on the surface of the Earth. Their high level of detail makes them extremely valuable for land-cover analysis. Unfortunately, just like aerial image data from current photogrammetric missions, they are characterized by a much lower spectral resolution compared to satellite data.

The solution to the problem of obtaining high-quality image data from one platform is the process of fusing images from different sensors [3]. Multi-sensor data fusion is the process of integrating data from different sensors to obtain a composite image which, due to its greater information capacity, is conducive to analyses of the photographed land cover [4]. The resulting enhanced images are used in the main remote sensing processes: object identification, land-cover classification, and change detection. Pansharpening methods are widely used for the fusion of remote sensing images. The goal of pansharpening is to integrate high-spatial-resolution data with high-spectral-resolution data. The output image of this process would ideally have the same spatial characteristics as the high-spatial-resolution input image and the same spectral characteristics as the high-spectral-resolution input image. However, it is known that the fusion process results in a partial loss of this information.

When data from different sensors are combined, more factors adversely affect the quality of the sharpened image compared to integrating data from the same platform. There are four factors with a fundamental impact on the quality of the enhanced images. They are all critical in the process of fusion of satellite and aerial images. Firstly, when working with data from different sensors, it is highly probable that images will be acquired at different times. Due to this fact, changes may occur in the studied area related to the vegetation period of plants, illumination conditions, natural disasters, or anthropogenic activity, which in turn increases the spectral distortion of the enhanced image [2]. It has been demonstrated that, when combining high-resolution UAV (Unmanned Aerial Vehicle) and multispectral satellite data, the difference for natural areas should not exceed two weeks [5]. Furthermore, the resolution ratio (RR), i.e., the difference between the GSD (Ground Sampling Distance) values of the input data, should be taken into account. For data from the same platform, the ratio should not exceed 1:5 [6].

In contrast, for data from different sensors, the ratio may be much higher, e.g., for aerial panchromatic data and multispectral satellite data (Sentinel-2), it may amount to 1:70. However, as the authors’ previous research has shown [7,8,9], with a ratio higher than 1:5, it is also possible to obtain a product that can improve terrain analysis compared to the interpretation of two separate products in the form of an aerial photo with a high spatial resolution and satellite imagery with a high spectral resolution. Another aspect is the co-registration of data. The results of research conducted by Blanc et al. [10] indicated that the standard deviation of geometric distortion values of 0.1 pixels already has a noticeable influence on the quality of the enhanced image. The last factor is the mutual coverage of the spectral input ranges in the pansharpening process. A larger spectral coverage between the panchromatic and multispectral bands results in a lower spectral distortion of the sharpened image.

No known fusion method can eliminate the impact of the factors mentioned above. The authors did not find any known universal method that would be appropriate regardless of the type of input data and the use of enhanced images [1,2,11]. Compared to research conducted on data from the same altitude, very few studies attempted to find a method of integrating images from different altitudes in order to produce enhanced images of the highest possible spectral quality while maintaining high spatial resolution. The high quality of the enhanced image is extremely important as it favors advanced qualitative and quantitative analyses of the environment, including land-cover classification [2,12]. For this purpose, methods of combining UAV and satellite optical data [5,8,13,14], methods of integrating data obtained from aircraft with satellite data [15,16], or methods of combining optical and radar satellite data [17,18,19,20] have been proposed. In their studies [7,21,22], the authors proved the increase in the interpretation potential of archival single-band aerial photographs as a result of combining these data with archival satellite images.

All of the mentioned studies focused on the issue of increasing the interpretation or classification potential of the enhanced image through an improvement in its spectral quality. However, the studies were carried out with reference to the entire obtained image showing different types of land cover or fragments of the image on which a group of objects with similar features—e.g., natural and artificial objects—was photographed, without taking into account the individual features of the objects. In [9], the authors proved that a panchromatic image, which is appropriate for maintaining high spectral quality in the context of the entire analyzed image, is not always appropriate for best maintaining the spectral reflectance characteristics of the individual types of the photographed land cover. This article describes the research carried out to solve this problem.

## 2. Purpose of the Study

The purpose of the study was to develop a methodology for increasing the spectral quality of images enhanced by the fusion of data from different sensors. The authors aimed to obtain, in the process of pansharpening of satellite images and aerial photographs, a resulting product of higher spectral quality than that using the original aerial panchromatic band as high-spatial-resolution data. Satellite images constituted data of high spectral resolution, and aerial photographs provided data of high spatial resolution. The purpose of the study was achieved by developing an innovative method which involved simulating a new image with a spatial resolution equal to that of the original aerial panchromatic image.

When the high-resolution panchromatic band is available, the choice of pansharpening method is one of the most important issues. However, when integrating high-resolution RGB image with multispectral data and the panchromatic band is not available, the simulation of the panchromatic band is crucial before application of a data fusion algorithm. Therefore, panchromatic channel simulation is a common problem. One of the simplest methods of panchromatic band simulation is the average of the high-resolution band in the multispectral image, i.e., the average of R, G, B, and NIR (near-infrared) bands [23]. When combining different data types, calculation of the arithmetic mean of all multispectral or hyperspectral bands covering the spectrum of available multispectral bands is a common practice [24]. Another approach is based on the separate application of simulated high-resolution bands for the visible and near-infrared range [25]. Other studies used the fact that spectral reflectance coefficients of similar terrain objects are practically invariable in two spectral ranges, and the simulation of missing high-resolution bands was based on that relationship [26].

The novelty presented herein was the assumption that, for individual objects—types of land cover identified on image data (aerial and satellite)—functional dependencies which are necessary to generate a new high-spatial-resolution band were developed. Functional dependencies express the relationship between the aerial panchromatic image and aerial multispectral images: red, green, and blue. The resulting new high- spatial-resolution image is combined using the pansharpening method with multispectral satellite imaging. It was expected that the image generated via the fusion process would have less spectral distortion than the sharpened image due to the integration of the original aerial panchromatic image and the same satellite data. The proposed new method of integrating data from different sensors not only yields images of higher spectral quality, but it also solves the problem of some platforms, e.g., Sentinel-2, not recording the panchromatic band. It should also be noted that the presented method applies not only to aerial multispectral data acquired during current photogrammetric missions, but also to archival aerial multispectral images obtained in the red, green, and blue bands as long as there is appropriate archival satellite multispectral imagery.

## 3. Data and Preprocessing

**Figure 1:**
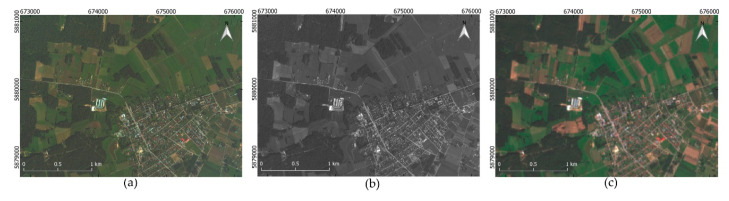
The data used for the research: (**a**) aerial MS (multispectral) image, (**b**) aerial PAN (panchromatic) image, and (**c**) satellite MS imagery.

This article used imagery data showing the eastern part of the Podlaskie province of Poland, covering the town of Michałowo and the areas located to the northwest of the town. The study area was selected due to the availability of aerial images and free-of-charge satellite images with a low time shift, as well as the high variety of the forms of land cover (Figure 1).

This study used panchromatic and multispectral aerial images (in the blue, green, and red range) recorded with a digital mapping camera (DMC) II 230 provided by MGGP Aero and multispectral satellite images recorded by the Sentinel-2 satellite, retrieved from https://scihub.copernicus.eu. The digital mapping camera (DMC) is a digital large-format camera equipped with five nadir-looking camera heads. It includes four multispectral cameras in red, green, blue, and near-infrared ranges and one high-resolution panchromatic camera head (Table 1). Each multispectral camera includes a CCD (Charge Coupled Device) with 7.2 µm pixel size and 45 mm focal length. The panchromatic camera has a CCD with 5.6 µm pixel size and 92 mm focal length [27,28]. The multispectral medium-resolution source of imagery data was the European Union Copernicus satellite Sentinel-2 [29,30]. The multispectral sensor mounted on Sentinel-2 platform provides 10 m, 20 m, and 60 m multispectral data in a wide range of the electromagnetic spectrum, from the visible range to short-wavelength infrared (SWIR). The Sentinel-2 constellation provides optical imagery of the worldwide land surface with a 5 day revisit period [31]. The aerial data were acquired on 20 August 2016, while the satellite imagery was recorded on 28 August 2016. The spatial resolution of the aerial images was 0.3 m. Four satellite bands with a spatial resolution of 10 m (bands 2–4 and 8 of Sentinel-2) and six bands with a resolution of 20 m (bands 5–7, 9, and 11–12 of Sentinel-2) were used in the study.

Table 1 shows the spectral reflectance characteristics of the aerial sensor (DMC II 230) and the satellite sensor (S2A) on the Sentinel-2 platform, showing the differences in the sensitivity of these sensors.

Both satellite and aerial data were geometrically corrected. The geometric correction process included orthorectification, spatial registration in the UTM/WGS84 (Universal Transverse Mercator/World Geodetic System ’84) projection, and image co-registration. Satellite images carrying information on DN (digital number) values were calibrated using the coefficients provided by the manufacturer to obtain top-of-atmosphere (TOA) reflectance values. These images were then subjected to atmospheric correction with the Quick Atmospheric Correction algorithm [32].

## 4. Methodology

**Figure 2:**
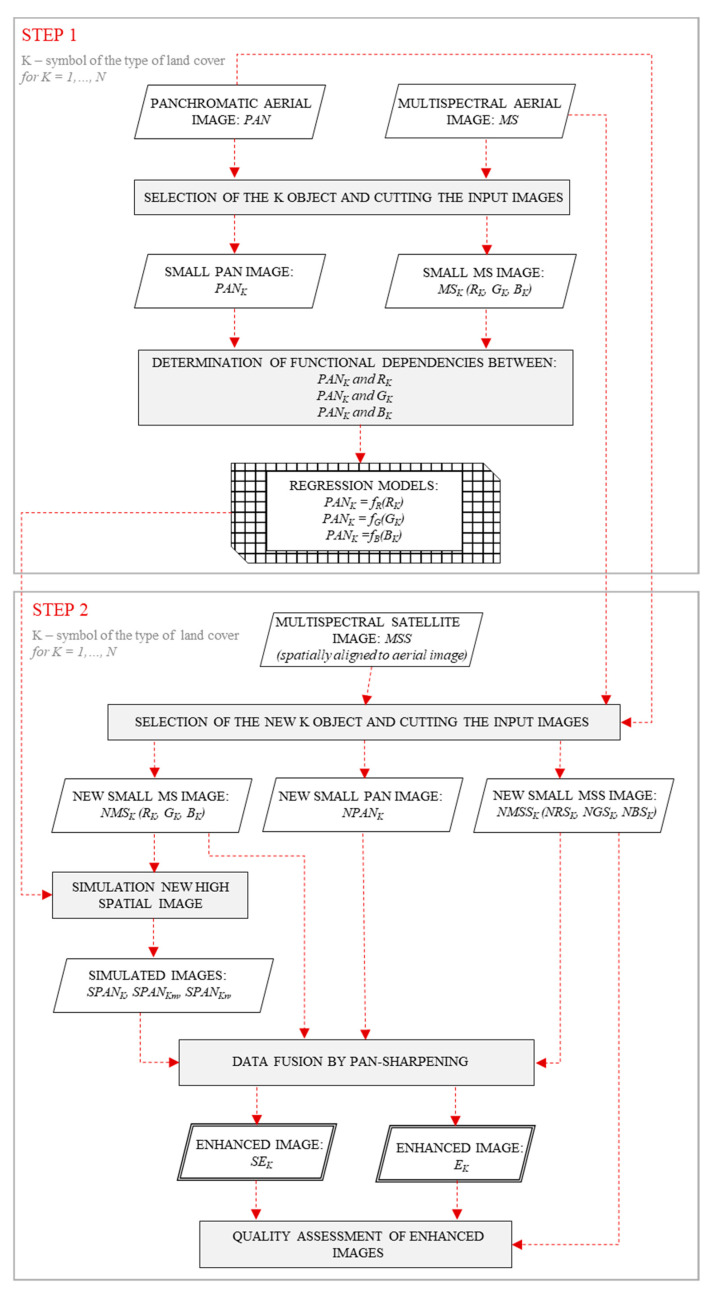
Flowchart for the data fusion.

The study consisted of two main stages (Figure 2). The first was a test stage which enabled the development of functional relationships between the aerial panchromatic band (PAN_K_) and aerial multispectral bands (R_K_, G_K_, and B_K_) for each studied class of land cover. These relationships were determined for several samples selected manually—types of land cover photographed by an aerial sensor. Natural objects (forests, meadows, fields, and bare soils) were selected for the study (Appendix A). The surface area of the samples was not less than 200 m^2^ (not less than 2800 pixels) [33,34,35]. The determination of mathematical functions describing the relationships between the pixel values of two images, PAN_K_ and individual MS_K_ bands, was based on regression models (Equations (1)–(3)).

PAN_K_ = f_R_(R_K_), (1)

PAN_K_ = f_G_(G_K_), (2)

PAN_K_ = f_B_(B_K_), (3)

where K is the type of land cover (K = 1, …, *N*).

The parameters of the regression models were estimated using the least-squares method. The regression model, which describes the relationships between empirical data, was selected as a result of evaluating the coefficients of determination (*R^2^*).

**Figure 3:**
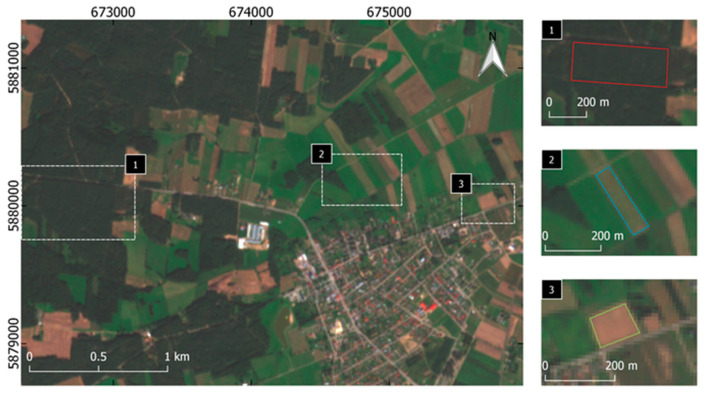
Locations of samples (red for forest, blue for mown meadow, and green for bare soil).

In the second stage, new high-spatial bands based on the previously developed functional relationships were simulated. The process of combining high-spatial-resolution data with high-spectral-resolution data was carried out, and the spectral quality of the resulting enhanced images was evaluated. Work started with the detection of objects belonging to the same land-cover classes that were used for tests but located at different points in the images. Isolation of individual classes of objects was based on the analysis of texture and digital numbers of pixels. Objects, for which no changes were observed over the time (i.e., no difference occurred between the acquisition of the aerial and satellite images), were selected for the study. In this way, sections of aerial and satellite images with surface areas of at least 7000 m^2^ were manually obtained (Figure 3).

The size of these sections of images was significantly limited by the dimensions of the fields of land. Using an original approach, new bands with high spatial resolution were simulated separately for each class of land cover. For this purpose, the aerial multispectral image (red, green, and blue bands) and the formulas established within the first stage were applied. The new image was generated through three procedures. The first consisted of summing up three components, each of which expressed the pixel values of the aerial panchromatic band as a function of the pixel values of individual aerial multispectral bands, i.e., red, green, and blue (Equation (4)). The second procedure was to average these three components (Equation (5)), and the third was to weigh the components on the basis of coefficients used by the National Television System Committee (NTSC) in the YUV color space (Equation (6)). The values of the weights refer to the perception capacity of humans [36].

SPAN_K_ = f_R_(R_K_) + f_G_(G_K_) + f_B_(B_K_). (4)

(5) $${\mathrm{SPAN}}_{\mathrm{Km}}\;=\;\frac{\left[f_{R}\left(R_{K}\right)+\;f_{G}\left(G_{K}\right)+\;f_{B}\left(B_{K}\right)\right]}{3}.$$

SPAN_Kn_ = 0.299∙f_R_(R_K_) + 0.587∙f_G_(G_K_) + 0.114∙f_B_(B_K_). (6)

Then, the generated synthetic images of high spatial resolution (equal to the resolution of aerial data) and the multispectral satellite images were integrated for each of the tested objects. The data fusion was carried out using the Gram-Schmidt pansharpening method. This method was selected because of its speed and ease of implementation and its relatively good preservation of spectral information [37]. For comparison purposes, the original panchromatic and multispectral aerial images (red, green, and blue bands) were also integrated with the multispectral satellite images for each of the tested land-cover classes. The method known as “consistency” or “Wald’s protocol first property” [2,3] was used to assess the quality of the enhanced images. The spatial resolution of each composite image was degraded to the spatial resolution of the multispectral image. Statistical analysis was performed using five spectral quality assessment indicators: universal image quality index (UIQI), peak signal-to-noise ratio index (PSNR), correlation coefficient (CC), structural similarity (SSIM) index, and spectral angle mapper (SAM) [23,38,39,40,41]. The purpose of this evaluation was to determine the degree of spectral compatibility between the enhanced image degraded to the spatial resolution of the original satellite multispectral image and the original satellite multispectral image. Additionally, the spectral reflectance characteristics of the studied objects collected from enhanced images and satellite imaging were compared. A comparison of the high-spatial-resolution bands used in the fusion process in relation to their information amount was also made. For this purpose, the values of information entropy (Shannon entropy) were used. Information entropy is a measure of the occurrence of the randomness or uncertainty in an image or a signal, i.e., the information richness of an image or a signal. Shannon H (S) entropy is defined as follows [42,43,44,45]:

(7) $$H\left(S\right)=-\overset{n}{\underset{i=1}{\sum }}p\left(S_{i}\right)log_{2}p\left(S_{i}\right).$$

Concerning image analysis, *S_i_* denotes the pixel value and *p*(*S_i_*) denotes the probability of this value occurring in the image. A higher entropy value denotes higher image information content and, hence, higher image quality [43,46].

Nevertheless, it is known that the Shannon parameter cannot be used to quantify the spatial information (in terms of the configurational-spatial distribution of pixels and some of the compositional information, e.g., contrast) [47,48].

In order to measure the spatial information content of an image, Boltzmann entropy (BE) has been used relatively recently [48,49]. This parameter was first proposed in the 1870s to determine the configuration and composition of a thermodynamic system in physics. It is defined as follows (Equation (8)):

(8) $$S=k_{B}log\left(W\right),$$

where *S* is the Boltzmann entropy value for the given system, *W* is the number of distinguishable microstates of that system, and *k_B_* is a constant value [47]. An obstacle in the use of Boltzamn entropy, not only in thermodynamics, is the lack of a universal definition of a macrostate system and the need to develop a method for determining the number of microstates in the macrostate. In [48], a method for the application of Boltzmann entropy to images was proposed. The macrostate is defined here as an upscaled version of an original image generated by resampling this image with a 2 × 2 pixel window. At the same time, *W* is the number of all achievable results of the macrostate rescaling operation to the resolution of the original image with the assumption that all rescaling results have the same mean, minimum, and maximum values as the input data. Once *W* is determined, it becomes possible to find the relative entropy (or relative configurational entropy) using the Boltzmann equation (Equation (9)).

(9) $$S=k_{B}log\left(W\right)=log_{10}\left(W\right),$$

where *k_B_* = 1 [50]; the logarithm at base ten is used, while bases two and *e* are also allowed.

As a result of summing the relative entropies (calculated between two adjacent abstract levels), the absolute entropy is obtained. Using absolute entropy, it becomes possible to compare two images [48]. By using Boltzmann entropy, it is possible to quantify not only the statistical information but also the spatial information of a dataset. Moreover, it can be used as the correlation between spatial patterns and thermodynamic interpretations.

The absolute Boltzmann entropy of an image is a characterization of the uncertainty of downscaling from a single pixel to the original image. Given that uncertainty is the cornerstone of information theory, the relative and absolute Boltzmann entropies can serve as a basis for spatial information theory [51].

In [52], Boltzmann entropy was employed to quantify the spatial information of raster data, i.e., images, maps, digital elevation models, landscape mosaics, etc.

In spatial information theory, one of the fundamental issues is the measurement of information content. When fusing two images, the fundamental question is whether the information content of the fused image is greater than that of the original ones or not. Entropy is, by far, the most popular and widely accepted measure for information content [51].

Therefore, in our future research, we will deal with the spatial information aspect and the assessment of the degree of preservation of this information in the pansharpening process.

## 5. Results

**Figure 4:**
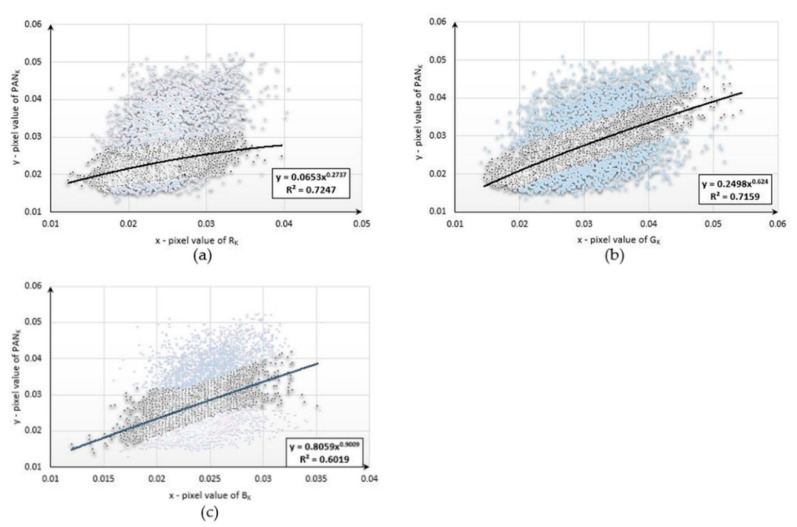
Diagrams presenting the functional relationships for the forest sample: (**a**) between PAN and red bands; (**b**) between PAN and green bands; (**c**) between PAN and blue bands.

**Figure 5:**
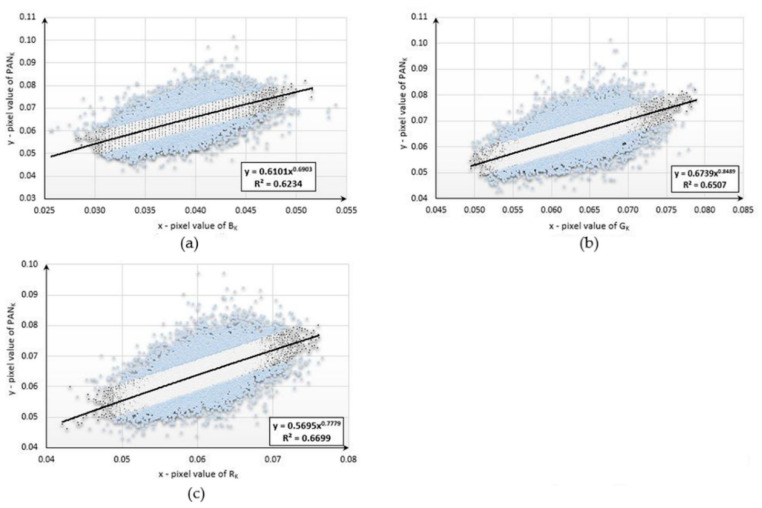
Diagrams presenting the functional relationships for the mowed meadow sample: (**a**) between PAN and red bands; (**b**) between PAN and green bands; (**c**) between PAN and blue bands.

**Figure 6:**
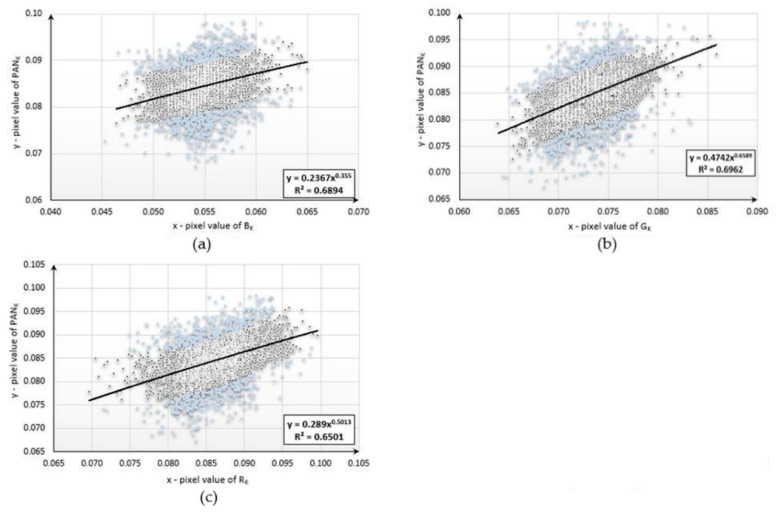
Diagrams presenting the functional relationships for the bare soil sample: (**a**) between PAN and red bands; (**b**) between PAN and green bands; (**c**) between PAN and blue bands.

This study began with the development of mathematical functions describing the relationships between the pixel values of the aerial panchromatic image (PAN_k_) and the aerial images obtained in the blue (B_k_), green (G_k_), and red (R_k_) ranges for each type of land cover studied. Below are diagrams (Figure 4, Figure 5 and Figure 6) presenting the functional relationships for three natural objects selected from the sample collection. For forest, the pixel number was 4977, for bare soil, the pixel number was 3588, and, for mowed meadow, the pixel number was 27,590. Before determining the function, the data were filtered on the basis of the value of the standard deviation. About 20% of the samples were rejected for each dataset.

The values of the coefficient of determination (*R^2^*) ranged from 0.6019 to 0.7247. The use of data filtering increased the accuracy of matching the designated functions; however, the values of the coefficient varied due to the number of samples used and the type of land cover for which the pixel values were not homogeneous. The functional relationships described above were then used to simulate new bands with high spatial resolution. For this purpose, new, corresponding fragments were selected from aerial and satellite images representing the same land-cover classes for which the mathematical functions were developed. Using the aerial multispectral image and mathematical functions, new synthetic images with a spatial resolution equal to that of the aerial image were generated for each of the objects under study. The simulated images were then integrated with the satellite imagery from the Sentinel-2 satellite. The original aerial images acquired in the blue, green, and red ranges were also combined with a satellite multispectral image. Several dozen enhanced images were generated following this procedure. Fragments of the three enhanced images are presented in Appendix B. The spectral quality of each of the samples—classes of natural land cover—was assessed. The results are presented in Table 2, Table 3 and Table 4.

In the case of the forest sample, the highest spectral quality was obtained when using the original aerial red band as the high-spatial-resolution data in the fusion process. Four out of five coefficients (excluding SAM) indicated an increase in the spectral quality of the image enhanced with the use of the aerial red band. The increase in quality, however, was insignificant in relation to the use of the original aerial panchromatic band.

The highest values of spectral quality coefficients for the mown meadow were obtained using a simulated SPAN_K_ band (Equation (4)). The highest increase in the value of the coefficients was recorded for bands 6–9 (vegetation red edge bands 6, 7, and 8a, as well as NIR band 8). The increase in spectral quality for the mown meadow sample was significant compared to the results obtained for the forest sample. Relative to the values obtained using the original panchromatic image in the fusion process, the values of the coefficients for the mown meadow sample in the case of the red edge and NIR bands increased from 8% to 14%.

The enhanced image of the highest spectral quality showing bare soil was obtained using SPAN_Kn_ (Equation (6)) as the high-spatial-resolution data in the pansharpening process. All the coefficients used clearly indicated an increase in the spectral quality of the enhanced image compared to the original aerial panchromatic image. For the bare soil sample, the highest increase in the values of the coefficients was observed for bands 5 (vegetation red edge), 11 (SWIR), and 12 (SWIR). For these bands, the values of the ratios increased relatively from 12% to 69%.

**Figure 7:**
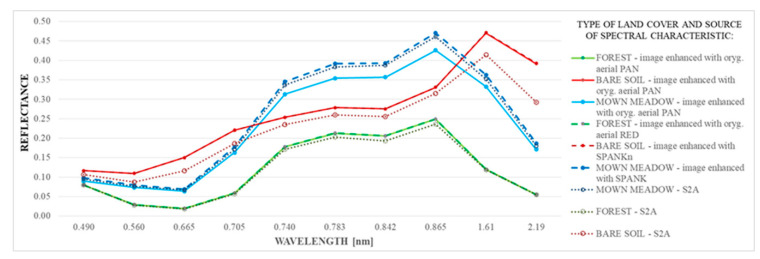
Spectral reflectance characteristics of the studied samples.

The spectral reflectance characteristics of the studied samples were also compared (Figure 7). Three diagrams were generated for each sample: the first one based on the sharpened image obtained from the original aerial PAN image, the second one based on the enhanced image with the highest spectral quality, and the third containing values obtained from the original Sentinel-2 multispectral image.

For the forest and bare soil samples, no difference was observed between the characteristics obtained from the enhanced images (both diagrams for each sample overlap). On the other hand, a noticeable improvement was observed for the mown meadow sample in bands corresponding to wavelengths of 0.705, 0.740, 0.783, 0.842, 0.865, 1.61, and 2.19 nm (bands 5–8a and 11–12 of Sentinel-2).

The entropy measure presented by Shannon can be used for measurements of the amount of information within an image [43,53]. In this paper, the entropy measure was used for the evaluation of information content [42] of simulated panchromatic bands used in the data fusion process. According to entropy theory, higher values indicate an image with richer detail (Table 5).

Obtained results showed that simulated panchromatic bands reached a higher information content than other potential panchromatic channels used in the pansharpening process, and, in the case of meadows, the information content was even increased.

## 6. Discussion and Conclusions

The process of combining images from different sensors is intended to obtain complete environmental information compared to a single image. This article presented a methodology for combining aerial and satellite data to improve the spectral quality of the sharpened image, thus meeting the expectations for the development of techniques of integrating data recorded by different sensors [4].

The research proved that in order to retain the highest possible spectral quality in the pansharpening process, it is necessary to simulate a new synthetic image with high spatial resolution, depending on the type of objects studied. The tests led to the development of a methodology for combining aerial data obtained with a DMC II 230 with Sentinel-2 satellite data for selected natural objects. The use of simulated high-spatial bands with high information content in the fusion process allowed the authors to obtain enhanced images with less spectral distortion than the original aerial panchromatic image for each sample (the type of land cover). This was confirmed by the values of the spectral quality assessment coefficients and by the spectral reflectance characteristics of the objects. However, the degree of spectral compatibility of the generated enhanced image characterized by the highest spectral quality and satellite multispectral imagery included in the integration process was different for the objects studied. In the case of the forest sample, the increase in spectral quality was negligible, whereas, for the remaining samples (mown meadow and bare soil), a significant increase in retained spectral information was found. The low values of the coefficients for the forest were probably due to the occurrence of tree shadows in the image, which could disturb the analysis. No method was found to simulate a new band with high spatial resolution or to select a suitable multispectral aerial band that would be universal for all objects studied. The essential difficulties (i.e., time-shift, resolution ratio, co-registration, differences in spectral resolution) of multi-sensor data fusion were described in Section 1. At the current stage of research, the main difficulty in applying the presented methodology is its degree of automation. The samples for the presented tests were selected manually, and the simulation of new channels and their fusion with satellite imagery were carried out separately for each of the tested land-cover types. This paper focused primarily on the essence of the simulation process of new high-spatial-resolution bands for the process of combining aerial and satellite images and the method of carrying out this process. In the next stage of the research, the authors will focus on the automation of the presented methodology by implementing segmentation and classification algorithms, so that the simulation process will be performed simultaneously for the entire image and not fragmentarily (for each sample separately). Additionally, the research will be extended to artificial objects.

Few studies are available concerning the fusion of aerial and satellite data. In the analyzed studies concerning the spectral quality of images enhanced by the integration of optical data from the same (such as [54,55,56]) or different platforms operating at the same altitudes (such as [57,58,59]), the entire images recorded by the sensor are subject to processing in the same way. The diversity of the types of land cover present in the image is not considered in the fusion process and the associated registration of different reflectance characteristics. Usually, the research involves checking which of the existing methods of pansharpening results in the highest quality of the enhanced image. Due to the differences in the degree of preservation of spectral information in the pansharpening process depending on the type of land cover, there is a need to take into account the spectral reflectance characteristics of the photographed objects in this process.

This article presented an innovative methodology of combining the optical data acquired from the satellite and aerial altitudes, which takes into account the individual spectral characteristics of the photographed objects by using new, simulated high-spatial-resolution bands or appropriate multispectral high-spatial-resolution bands. It is a methodology that applies both to multispectral aerial images acquired today and to archival aerial data registered in three spectral ranges (blue, green, and red), as long as there is an appropriate archival satellite multispectral images. The authors did not explore the usefulness of pansharpening methods in general, but rather focused on the role of the high-spatial-resolution band in the fusion process. Thanks to the developed methodology, the spectral information processed through pansharpening was retained to a higher degree than with the original panchromatic image. When combining data from different satellite platforms, the authors of [57,58] achieved high spectral quality of the enhanced images. For the best pansharpening methods applied, the values of spectral quality coefficients CC and SSIM were close to 1, while, for the worst methods, the CC values ranged from 0.1 to 0.9 and the SSIM values ranged from 0.1 to 0.4. In this study, mean CC values in the range of 0.3–0.9 and mean SSIM values in the range of 0.5–0.9 were obtained, depending on the object studied. However, when combining image data from such different platforms, it is expected that the resulting enhanced images will be encumbered with more spectral distortion than when combining data from the same platform. In previous studies [7,9,21], the authors proved that, despite the spectral distortions that occur, the enhanced images obtained by combining aerial and satellite data are useful for environmental analyses (interpretation and classification of land cover). In this article, the authors went one step further in developing the integration of aerial and satellite image data, and they presented the methodology for increasing the spectral quality of sharpened images, thus giving rise to the development of new techniques for combining images recorded at different altitudes.

## Figures and Tables

**Table 1:** The sensitivity of aerial (DMC II 230) and satellite sensors (S2A); the values for the aerial sensor were read from the camera calibration report and are approximate values.

DMC II 230					S2A				
Spectral Bands	FWHW ^1^ (nm)			Centre Wavelength (nm)	Spectral Bands	FWHW (nm)			Centre Wavelength (nm)
	Lower	Upper	Upper—Lower			Lower	Upper	Upper—Lower	
Blue	430	485	55	457.5	Blue	459.4	525.4	66	492.4
Green	505	560	55	532.5	Green	541.8	577.8	36	559.8
Red	600	665	65	632.5	Red	649.1	680.1	31	664.6
PAN	450	690	240	570.0	Vegetation Red Edge	696.6	711.6	15	704.1
The spectral range of the aerial panchromatic band mostly includes the spectral ranges of the blue, green, and red bands of Sentinel-2. For the other Sentinel-2 bands, there is no coverage. The aerial blue band spectrally covers the satellite blue band to some extent; mutual coverage between the red bands is present to a lesser extent. The aerial green band, on the other hand, partially covers the range of the blue band and the green band of the Sentinel-2 satellite.					Vegetation Red Edge	733	748	15	740.5
					Vegetation Red Edge	772.8	792.8	20	782.8
					NIR	779.8	885.8	106	832.8
					Vegetation Red Edge	854.2	875.2	21	864.7
					SWIR ^2^	1568.2	1659.2	91	1613.7
					SWIR	2114.9	2289.9	175	2202.4

^1^ full width at half maximum. ^2^ short-wave infrared.

**Table 2:** Spectral quality values for the forest sample (the best results are underlined).

Number of Sentinel-2 Band (Spatial Resolution (m))	Forest									
	UIQI		SSIM		PSNR		CC		SAM	
	Orig. Aerial PAN	Orig. Aerial Red	Orig. Aerial PAN	Orig. Aerial Red	Orig. Aerial PAN	Orig. Aerial Red	Orig. Aerial PAN	Orig. Aerial Red	Orig. Aerial PAN	Orig. Aerial RED
2 (10)	0.224	0.226	0.591	0.593	33.704	33.791	0.456	0.461	0.027	0.028
3 (10)	0.227	0.232	0.895	0.896	42.035	42.105	0.413	0.422		
4 (10)	0.243	0.248	0.948	0.949	45.409	45.481	0.430	0.440		
5 (20)	0.129	0.136	0.598	0.608	32.049	32.121	0.146	0.155		
6 (20)	0.113	0.121	0.218	0.226	22.470	22.542	0.129	0.138		
7 (20)	0.111	0.119	0.188	0.197	20.963	21.035	0.126	0.136		
8 (10)	0.227	0.231	0.316	0.319	25.172	25.259	0.427	0.434		
8a (20)	0.113	0.122	0.171	0.179	19.599	19.671	0.129	0.139		
11 (20)	0.123	0.131	0.319	0.328	25.859	25.930	0.140	0.149		
12 (20)	0.142	0.150	0.628	0.639	32.623	32.623	0.161	0.170		
Arithmetic Mean:	0.165	0.172	0.487	0.493	29.988	30.056	0.256	0.264		

**Table 3:** Spectral quality values for the mown meadow sample (the best results are underlined).

Number of Sentinel-2 Band (Spatial Resolution (m))	Mown Meadow									
	UIQI		SSIM		PSNR		CC		SAM	
	Orig. Aerial PAN	SPAN_K_	Orig. Aerial PAN	SPAN_K_	Orig. Aerial PAN	SPAN_K_	Orig. Aerial PAN	SPAN_K_	Orig. Aerial PAN	SPAN_K_
2 (10)	0.87	0.93	0.90	0.94	33.1	35.1	0.88	0.92	0.072	0.040
3 (10)	0.89	0.93	0.91	0.95	35.4	37.4	0.89	0.93		
4 (10)	0.91	0.94	0.94	0.96	37.5	39.1	0.91	0.94		
5 (20)	0.78	0.85	0.80	0.86	25.7	27.3	0.79	0.85		
6 (20)	0.74	0.84	0.75	0.84	18.8	20.9	0.75	0.84		
7 (20)	0.73	0.84	0.74	0.84	17.4	19.6	0.74	0.84		
8 (10)	0.82	0.90	0.82	0.90	19.3	21.6	0.83	0.90		
8a (20)	0.74	0.84	0.74	0.84	15.9	18.1	0.74	0.84		
11 (20)	0.78	0.85	0.78	0.85	19.5	21.1	0.79	0.85		
12 (20)	0.79	0.85	0.80	0.86	25.4	27.0	0.79	0.85		
Arithmetic Mean:	0.80	0.88	0.82	0.88	24.8	26.7	0.81	0.87		

**Table 4:** Spectral quality values for the bare soil sample (the best results are underlined).

Number of Sentinel-2 Band (Spatial Resolution (m))	Bare Soil									
	UIQI		SSIM		PSNR		CC		SAM	
	Orig. Aerial PAN	SPAN_Kn_	Orig. Aerial PAN	SPAN_Kn_	Orig. Aerial PAN	SPAN_Kn_	Orig. Aerial PAN	SPAN_Kn_	Orig. Aerial PAN	SPAN_Kn_
2 (10)	0.61	0.64	0.99	0.99	54.0	54.4	0.79	0.84	0.023	0.022
3 (10)	0.60	0.65	0.96	0.99	48.0	48.7	0.68	0.78		
4 (10)	0.62	0.66	0.87	0.93	41.3	42.4	0.75	0.87		
5 (20)	0.46	0.55	0.79	0.89	32.1	38.8	0.57	0.66		
6 (20)	0.55	0.57	0.85	0.87	32.7	39.3	0.63	0.67		
7 (20)	0.56	0.56	0.80	0.82	30.3	36.5	0.62	0.64		
8 (10)	0.62	0.67	0.90	0.93	44.7	46.5	0.82	0.89		
8a (20)	0.56	0.58	0.79	0.81	29.6	36.1	0.63	0.65		
11 (20)	0.30	0.47	0.52	0.66	23.4	31.0	0.36	0.60		
12 (20)	0.44	0.57	0.55	0.67	21.8	29.0	0.53	0.68		
Arithmetic Mean:	0.53	0.59	0.80	0.86	35.8	40.3	0.64	0.73		

**Table 5:** Entropy values for original aerial panchromatic band, new simulated (the best results are underlined).

Panchromatic Band	Forest	Mown Meadow	Bare Soil
orig. aerial PAN	3.1551	2.8360	2.4999
orig. aerial blue	2.0270	1.7048	1.3274
orig. aerial green	2.1597	2.0589	1.4207
orig. aerial red	2.5023	2.3871	1.6698
SPAN_K_	2.2088	3.1394	1.8792
SPAN_Km_	2.2780	2.8125	1.8269
SPAN_Kn_	2.3294	2.9678	1.9965

**Figure A1:**
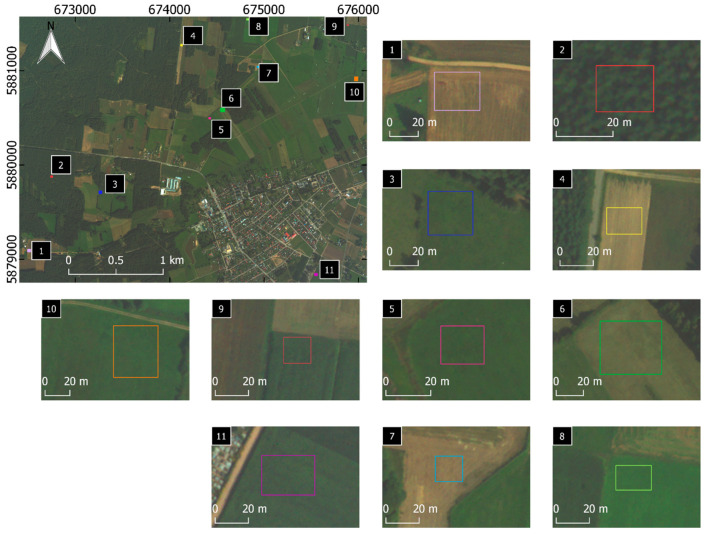
Locations of samples used for the study (1, 4, and 7—bare soils; 2—forest; 3,5,8, and 10—meadows; 6—mowed meadow; 9 and 11—farmlands).

**Figure A2:**
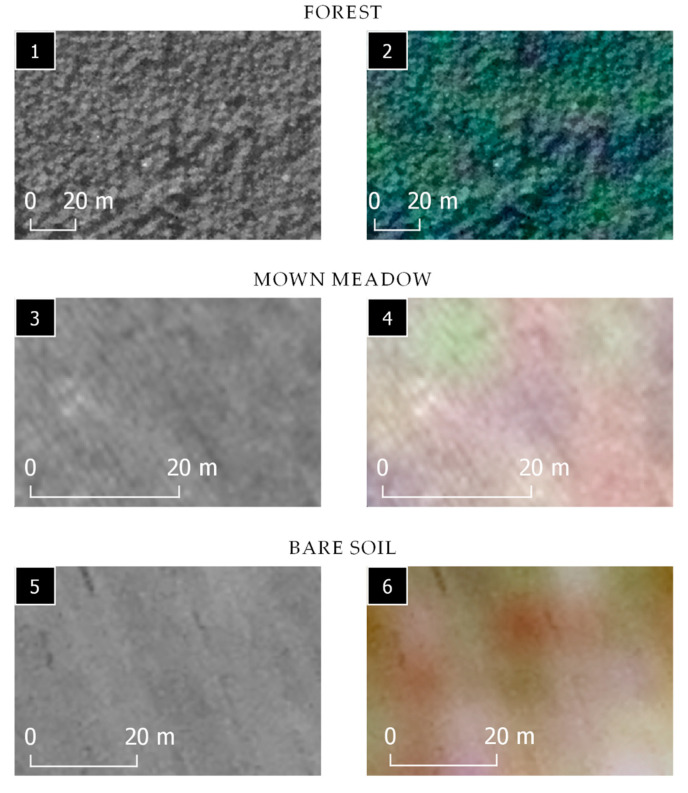
1, 3, and 5—samples of the best high-spatial images; 2, 4, and 6—samples of images in natural color composition enhanced with the best high-spatial-resolution image.
